# MiR-187 Targets the Androgen-Regulated Gene *ALDH1A3* in Prostate Cancer

**DOI:** 10.1371/journal.pone.0125576

**Published:** 2015-05-13

**Authors:** Irene Casanova-Salas, Esther Masiá, Ana Armiñán, Ana Calatrava, Caterina Mancarella, José Rubio-Briones, Katia Scotlandi, Maria Jesús Vicent, José Antonio López-Guerrero

**Affiliations:** 1 Laboratory of Molecular Biology, Fundación Instituto Valenciano de Oncología, Valencia, Spain; 2 Polymer Therapeutics Laboratory, Centro de Investigación Príncipe Felipe, Valencia, Spain; 3 Department of Pathology, Fundación Instituto Valenciano de Oncología, Valencia, Spain; 4 Laboratory of Experimental Oncology, Istituto Ortopedico Rizzoli, Bologna, Italy; 5 Department of Urology, Fundación Instituto Valenciano de Oncología, Valencia, Spain; Innsbruck Medical University, AUSTRIA

## Abstract

miRNAs are predicted to control the activity of approximately 60% of all protein-coding genes participating in the regulation of several cellular processes and diseases, including cancer. Recently, we have demonstrated that miR-187 is significantly downregulated in prostate cancer (PCa) and here we propose a proteomic approach to identify its potential targets. For this purpose, PC-3 cells were transiently transfected with miR-187 precursor and miRNA mimic negative control. Proteins were analyzed by a two-dimensional difference gel electrophoresis (2D-DIGE) and defined as differentially regulated if the observed fold change was ±1.06. Then, MALDI-TOF MS analysis was performed after protein digestion and low abundance proteins were identified by LC–MS/MS. Peptides were identified by searching against the Expasy SWISS PROT database, and target validation was performed both in vitro by western blot and qRT-PCR and in clinical samples by qRT-PCR, immunohistochemistry and ELISA. DIGE analysis showed 9 differentially expressed spots (p<0.05) and 7 showed a down-regulated expression upon miR-187 re-introduction. Among these targets we identified aldehyde dehydrogenase 1A3 (ALDH1A3). ALDH1A3 expression was significantly downregulated in PC3, LNCaP and DU-145 cells after miR-187 re-introduction. Supporting these data, the expression of ALDH1A3 was found significantly (p<0.0001) up-regulated in PCa samples and inversely correlated (p<0.0001) with miR-187 expression, its expression being directly associated with Gleason score (p = 0.05). The expression of ALDH1A3 was measured in urine samples to evaluate the predictive capability of this biomarker for the presence of PCa and, at a signification level of 10%, PSA and also ALDH1A3 were significantly associated with a positive biopsy of PCa. In conclusion, our data illustrate for the first time the role of ALDH1A3 as a miR-187 target in PCa and provide insights in the utility of using this protein as a new biomarker for PCa.

## Introduction

Prostate cancer (PCa) is the most common cancer and the second leading cause of cancer death in men [1]. Approximately one in three men over the age of 50 years shows histological evidence of PCa. However, only 10% of these will be correctly diagnosed with clinically significant PCa [2]. PSA levels combined with digital rectal examination (DRE) are the main criteria for PCa diagnosis, but often lead to over diagnosis and overtreatment [3]. Consequently, the identification of new biomarkers, able to improve the diagnosis and detection of potentially aggressive PCa, are needed to better support clinical decisions.

miRNAs are a class of small non-coding RNA molecules consisting of 19–22 nucleotides; they are involved in a variety of biological processes, including development, differentiation, apoptosis and cell proliferation. miRNAs regulate gene expression through translational repression and mRNA cleavage of more than 60% of protein coding genes [4, 5]. Several studies suggest that an individual miRNA can regulate hundreds of targets [6] and can function either as a tumor suppressor or oncogene, depending on the target genes [7], as well as contributing to the initiation and development of various types of cancer, including PCa [5].

Using miRNA microarray analysis (NCBI Gene Expression Omnibus database accession number GSE45604), our group identified miR-187 as a tumor suppressor miRNA in PCa [8]. Although its utility has been demonstrated in the diagnostic setting, to date no experimentally confirmed targets for miR-187 have been identified in PCa. Most computational algorithms predict miRNA targets based primarily on sequence complementarities between the 5’ end of the mature miRNA and the 3’-untranslated region (3’-UTR) of target mRNAs; however, these algorithms yield relatively high rates of both false positives and false negatives [6]. Moreover, it is known that more than 25% of experimentally validated targets cannot be predicted by any of the most common miRNA target prediction software [9]. Therefore, gene expression and proteomic screening approaches are urgently needed to experimentally identify miRNA targets.

This study used a proteomic approach based on two-dimensional gel electrophoresis (2D-DIGE) followed by matrix-assisted laser desorption-ionization time-of-flight mass spectrometry (MALDI-TOF MS) analysis and, for the first time, identified *ALDH1A3* as a miR-187 target in PCa. In addition, the potential utility of ALDH1A3 as a tumor biomarker was evaluated.

## Material and Methods

### 2.1. Clinical prostate specimens

Formalin-fixed and paraffin-embedded (FFPE) blocks corresponding to 195 PCa patients were retrieved from the archives of the Biobank of the *Fundación Instituto Valenciano de Oncología* accomplishing the following inclusion criteria: specimens obtained from radical retropubic prostatectomies between 1996 and 2002 and no history of previous treatment for PCa (including androgen deprivation therapy or chemotherapy prior to surgery). All patients gave written informed consent for tissue donation for research purposes before tissue collection, and the study was approved by the Ethics Committee of the Fundación Instituto Valenciano de Oncología (ref. number. 2010–19). Exclusion criteria included any previous treatment or presence of other tumors together with the unacceptance of donation consent. The clinical data were reviewed from the clinical records and stored in a PCa-specific database. Patient characteristics and demographics are shown in Table 1. Gleason score was uniformly assessed by the same pathologist (AC). For comparative and calibration purposes, 8 samples of normal prostate tissue obtained from patients undergoing radical cystectomies without pathological evidence of prostatic disease were also analyzed.

**Table 1:** Demographics and main clinical and pathological features of the analyzed series.

Parameters	Retrospective series	
	n	%
**PSA**		
<10ng/ml	109	**55.8**
10–20ng/ml	54	**27.7**
>20ng/ml	32	**16.5**
**Gleason-sp**		
≤6	66	**33.8**
7	106	**54.4**
8–10	23	**11.8**
**cT**		
<cT2b	177	**90.8**
cT2b	18	**9.2**
**pT**		
<pT2	91	**46.7**
≥pT3	104	**53.3**
**pN** *		
pN0	169	**93.9**
pN≥1	11	**6.1**

SP, specimen; cT, clinical stage; pT, pathological stage; PSA, prostatic specific antigen; pN, pathologic stage with respect to lymph node status; NA, not available.

* Lymphadenectomy was limited to the obturator fossa in most of the cases at the inclusion period.

Ten fresh tissue samples from histologically confirmed PCa were retrieved from the archives of the biobank from Hospital Clínico Universitario de Valencia (INCLIVA) for validation purposes. Total urine samples were obtained after DRE and immediately prior to diagnostic needle biopsy from an independent cohort of 123 men with suspicion of PCa, from whom 63 lead to a positive biopsy.

### 2.2. Cell lines and miRNA transfection

PC-3, LNCaP, DU-145, and 22RV1 were cultured in RPMI 1640 (GIBCO, Invitrogen, Life Technologies, CA, USA) while vCaP PCa-derived cells were cultured in DMEM (ATCC, Middlesex, UK) medium, with 10% fetal bovine serum, 100U/ml Penincillin and 0.1ug/ml Streptomycine at standard cell culture conditions (37°C in 5% CO_2_ in a humidified incubator). miR-187, which has previously been found to be downregulated in PCa [8], was analyzed in these cell lines by qRT-PCR as described below.

PC-3, LNCaP and DU-145 cells were transiently transfected, using siPORT NeoFX Transfection Agent (Applied Biosystems, Life Technologies, California, USA), with 40nM precursors of miR-187 (hsa-miR-187-3p miRNA mimic) and miRNA mimic negative control 1#, according to the manufacturer’s protocol (Applied Biosystems, Life Technologies, California, USA). Cells were harvested 72 h after transfection and cell viability was measured to evaluate its toxicity using the CellTiter 96 AQueous nonradioactive cell proliferation assay (Promega, Wisconsin, USA) in accordance with the manufacturer’s instructions.

### 2.3. Identification of target genes for miR-187

Proteins from the miR-187 mimic versus miRNA mimic negative control 1# transfected PC-3 cells were analyzed by two-dimensional difference gel electrophoresis (2D-DIGE). Briefly, proteins of the two compared groups were precipitated using the 2-D Clean-Up kit (GE Healthcare, Piscatawey, NJ, USA). Samples were then resuspended in DIGE staining buffer DIGE (7M urea, 2 M tiourea, 4% CHAPS, 20 mM Tris) and quantified using Bradford protein assay (BioRad, Hercules, CA, USA). Samples were labelled with 400 pmol/50 μg of protein with the CyDye DIGE Fluor fluorophors Cy3 and Cy5 (GE Healthcare, Piscatawey, NJ, USA) as recommended by the manufacturer. A pool containing equal amounts of all samples was also prepared and labelled with Cy2 to be used as an internal standard on all gels to aid image matching and cross-gel statistical analysis. Six biological repeats of each transfected sample were performed and six gels were generated in total. Protein separation was performed by bidimensional electrophoresis. In the first dimension, proteins were separated according to their isoelectric point on immobilised 24 cm linear pH gradient (IPG) strips (GE Healthcare, Piscatawey, NJ, USA), rehydrated in rehydration buffer (7 M urea, 2 M thiourea, 4% CHAPS, 0.5% IPG Buffer, 50 mM DTT) overnight. In the second dimension, the proteins were separated according to the molecular weight in 25 cm x 21 cm x 1 mm 12.5% acrylamide gels. The gels were scanned with a Typhoon 9400 Variable Mode Imager (GE Healthcare, Piscatawey, NJ, USA) and the subsequent gel images were imported into the DeCyder Differential Analysis Software. Proteins were defined as differentially regulated if the observed fold change was ±1.06 (*p* < 0.05) between miRNA mimic negative control 1# transfected PC-3 and miRNA-187 mimic transfected PC-3. Protein digestion was performed with sequencing grade trypsin (Promega, Wisconsin, USA) as described elsewhere [10]. MALDI-TOF MS analysis was then performed using 0.5 μL of digestion mixture spotted onto the MALDI target plate. After air-drying the droplets at room temperature, 0.5 μL of matrix [5 mg/mL CHCA (Sigma, St.Louis, MO, USA) in 0.1% TFA-ACN/H2O (1:1, v/v)] was added and allowed to air-dry at room temperature. One known sample was processed identically as quality control. The resulting fractions were analyzed in a 4700 Proteomics Analyzer (ABSciex, Framingham, MA, USA) in positive reflectron mode (2000 shots each position). Low abundance proteins were identified by LC–MS/MS using a trap column (NanoLC Column, 3μ C18-CL, 75 μ m x 15cm, Eksigen, Dublin, CA, USA) through an isocratic flux of 0.1% TFA at 2 μ L/min during 10 min. Once peptides were concentrated into the pre-column they were eluted into the analytical column (LC Column, 3 μ C18-CL, 75um x 25cm, Eksigen, Dublin, CA, USA) to separate. Peptides were finally eluted using a 5a 40% B gradient over 30 min, into a nanoESI qQTOF mass spectrometer (5600 TripleTOF, ABSciex, Framingham, MA, USA). The information from the MS and MS/MS was analyzed with the Paragon algorithm, Protein Pilot Software (ABSciex, Framingham, MA, USA). The peptides were identified using the information in the tandem mass spectra by searching against the Expasy SWISS PROT database.

### 2.4. Western Blotting of ALDH1A3

For in vitro validation purposes, transfected PC-3, LNCaP and DU-145 cells with miR-187 mimic or miRNA mimic negative control (72 h) were harvested, rinsed with PBS and then lysed with ice-cold lysis buffer (50 mM TrisHCl pH = 8, 150 mM NaCl, 0.02% NaN_3_, 0.1%, Sodium dodecyl sulphate (SDS), 1% Nonidet P-40 (NP40), 0.5% deoxycholic acid (DOC), Protease inhibitor cocktail tablets (1 ×). Cell lysates were centrifuged at 10,000 rpm for 10 min at 4°C, the supernatant was then mixed with 5 × SDS sample buffer, boiled for 5 min and separated through 12% SDS-PAGE gels. After electrophoresis, the proteins were transferred to nitrocellulose membranes by electrophoretic transfer. The membranes were blocked in 5% skimmed milk for 1 h, rinsed and incubated overnight at 4°C with the primary antibodies: ALDH1A3 (Novus Biologicals, Littleton, CO, USA; 1:500 dilution) and β-actin (Millipore, Billerica, MA, USA; 1:200000 dilution). Excess antibody was then removed by washing the membrane in PBS/0.1% Tween 20, and the membranes were incubated for 1 h with horseradish peroxidase-conjugated secondary antibodies: goat anti-mouse IgG or donkey anti-rabbit IgG (1:10000) (GE Healthcare, Piscatawey, NJ, USA). After washes in PBS/0.1% Tween 20, immune-detection was performed using the enhanced chemiluminescent (ECL) Western blotting detection system (Euroclone, Milano, Italy), according to the manufacturer's instructions.

### 2.5. miRNA target reporter assay

PC-3 miR-187 mimic or Negative Control cells were transfected with 3’UTR Go Clone Reporter (50ng) (Switchgear Genomics, La Hulpe, Belgium) containing the 3’UTR sequence of ALDH1A3 cloned downstream of the RenSP luciferase reporter or the empty vector containing only the luciferase reporter using Dharmafect 2 reagent (Dharmacon, GE Healthcare,Piscatawey, NJ, USA). The cells were lysed and reporter activity was measured 24 h post transfection using LightSwitch Luciferase Assay Reagent (Switchgear Genomics).

### 2.6. RNA isolation and qRT-PCR

Isolation of RNA from both cell lines and clinical specimens was carried out using mirVana miRNA Isolation Kit (Ambion, Life Technologies, CA, USA). Total RNA was reverse transcribed using the TaqMan miRNA Reverse Transcription Kit and miRNA-specific stem-loop primers (Applied BioSystems, Life Technologies, CA, USA) and High Capacity cDNA Reverse Transcription Kit (Applied BioSystems, Life Technologies, CA, USA) according to the manufacturer’s indications. Then, 2 μl of this cDNA, corresponding to 96 PCa tumor samples, was amplified by real-time PCR in a final volume of 10 μl per reaction on an ABI 7500-fast thermocycler using mRNA assays (Applied Biosystems, Life Technologies, CA, USA). For miRNA evaluation, 1.33 μl of miRcDNA was amplified in a final volume of 20 μl. miRNA assays for RU44(001094), RU48 (001006) and miR-187 (001193), and mRNA assay for *ALDH1A3* (Hs00167476_m1) were used (Applied Biosystems, Life Technologies, CA, USA). All reactions were performed in triplicate. The relative expression of the mRNAs or miRNA was determined using the mean value of the control samples as calibrator and following the 2^-∆∆Ct^ method [11]. For cell line evaluations, miR-187 expression was analyzed by qRT-PCR using a universal human RNA pool (Cat# 740000 Stratagene, La Jolla, CA) as normalization control.

### 2.7. Immunohistochemistry of ALDH1A3

The same FFPE PCa blocks used for RNA analysis were incorporated in 11 tissue microarrays (TMA). Two or three representative areas (1 mm in diameter) of each tumor were selected for TMA production by first examining the hematoxylin & eosin-stained prostatectomy tumor slide and then sampling the tissue from the corresponding paraffin blocks. A tissue microarray instrument (Beecher Instruments, Sun Prairie, WI) was used for TMA assembly. From the TMA blocks, 3-μm-thick sections were subjected to immunohistochemical staining using rabbit anti-human ALDH1A3 polyclonal-Ab (Novus Biologicals, Littleton, CO, USA; 1:50 dilution). Human prostate tissue was used as positive control as recommended by the manufacturer. The percentage of ALDH1A3-positive cells and the cytoplasmic staining intensity were scored semiquantitatively, forming four groups (from 0 to 3). Cases were scored as low expressors when the staining intensity was between 0 and 1, and high expressors when the intensity was 2 and above.

### 2.8. ALDH1A3 ELISA

Urine samples from 123 patients with suspicion of PCa were centrifuged at 1000 x g to remove the debris. The urine supernatant was used to estimate the ALDH1A3 protein level by using a quantitative human sandwich ELISA kit (Blue Gene Biotech Co Ltd, Shanghai, China). The standard reference was between 0 and 50 ng/ml, with intra and inter-assay CV less than 10%. The optical density (O.D) was determined at 450nm using a Victor Multilabel Plate Reader (PerkinElmer Life Sciences, Massachusetts, USA).

### 2.9. Statistical analysis

To study the prognostic value of the *ALDH1A3* gene we used binary variables reflecting the positive status of measures. The association between *ALDH1A3* expression and clinicopathological parameters (categorical) was assessed using Spearman with significance considered at 5%. The impact of biological factors on BPFS and clinical PFS was determined by the Kaplan-Meier proportional risk log rank test [12]. Biochemical progression was defined as serum PSA greater than 0.4 ng/ml during follow-up and clinical progression was defined as local (prostatic fossa), regional (lymph nodes) or distant (metastasis) progression. BPFS and clinical PFS were considered individually from the date of surgery to the date of the event. Statistical analysis was done with SPSS, version 20.0. The Student's t test aplying FDR, p-value, PCA and hierarchical clustering were applied to the samples of the proteomic study. All results are given as mean ± SEM (GraphPad Prism 4.0 Software, Graph Pad Software, Inc.)

## Results

### 3.1. miRNA expression in PCa cell lines

**Fig 1:**
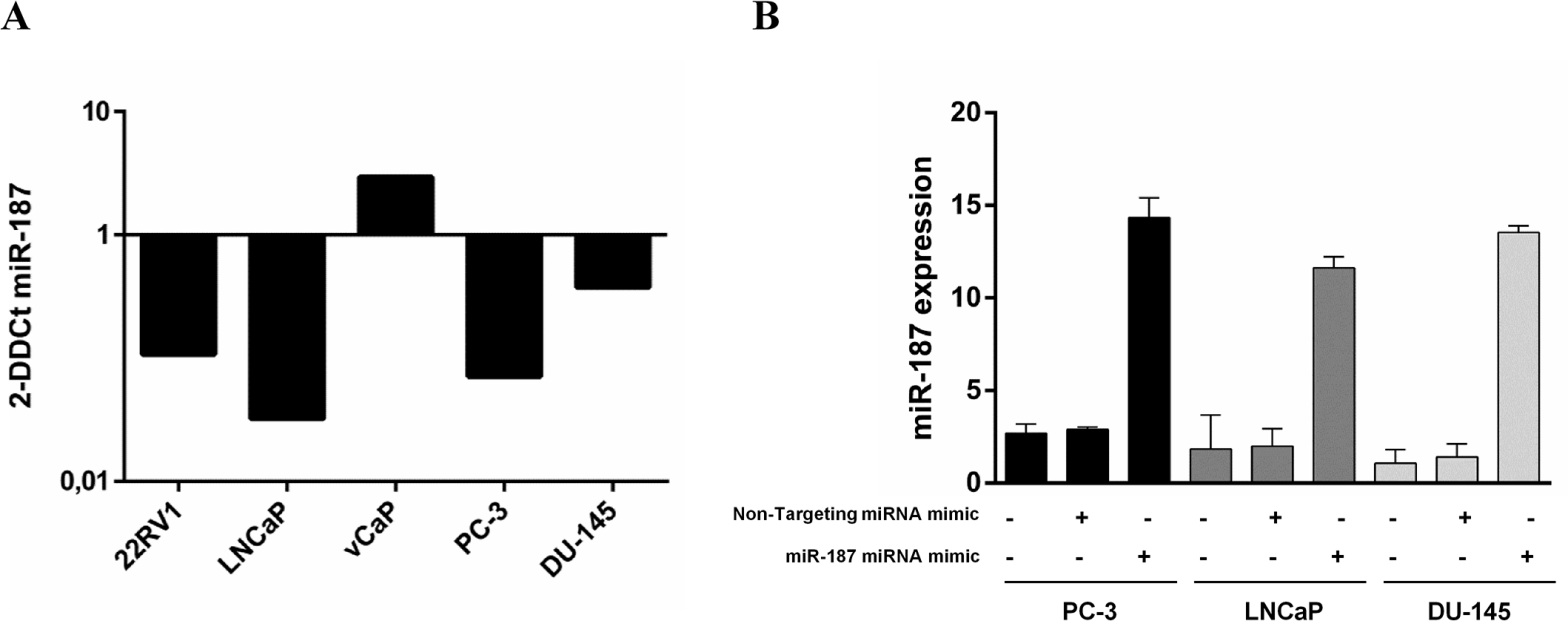
miRNA expression profile in PCa cell lines and miR-187 re-introduction. A) Expression of miR-187 was analyzed by qRT-PCR using a universal human RNA pool as calibrator to normalize the relative expression of the analyzed miRNAs following the 2^-∆∆Ct^ method. As can be appreciated, all cell lines but vCaP showed down-regulation of miR-187. B) miR-187 miRNA mimic and miRNA mimic negative control were transfected into PC-3, LNCaP and DU-145 cells for 72h. The re-introduction of miR-187 in PC-3, LNCaP and DU-145 was confirmed by real-time PCR. The histogram shows the increase in the miR-187 mRNA level in the miR-187 miRNA mimic transfected cells when compared with cells transfected with the miRNA mimic negative control and non-transfected cells.

Analysis of miRNA levels by qRT-PCR confirmed decreased expression of miR-187 in PCa cell lines: PC-3, LNCaP, DU-145 and 22RV1 (Fig 1A). In PC-3 the miR-187 expression level was equivalent to that observed previously in PCa patients [8], and for this reason this cell line was chosen for the subsequent analysis.

### 3.2. Identification of ALDH1A3 as putative miR-187 target

**Fig 2:**
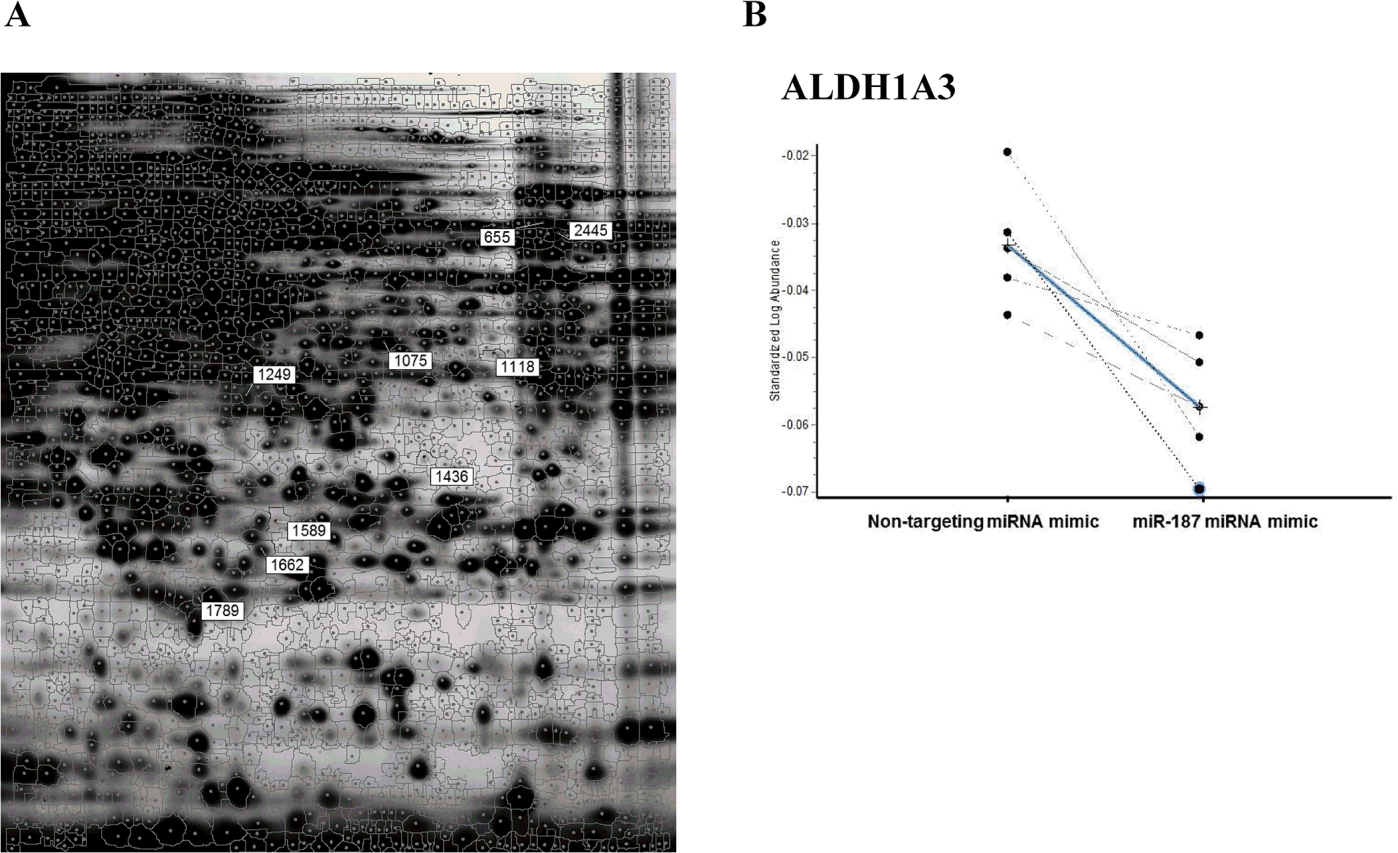
Identification of miR-187 putative targets by 2D-DIGE and LC-MS/MS. PC-3 cells were transfected either with miRNA mimic negative control or miR-187 miRNA mimic, harvested after 72h, and protein lysates were labeled with Cy3 or Cy5 (miR-187 and control) and Cy2 for the internal standard. A) 2D-DIGE gel image obtained at pH 3–10 and 12,5% SDS-polyacrilamide. The numbers refer to the identification given to the spots differentially expressed. Spot 655 was further identified by LC-MS/MS as ALDH1A3. B) Comparison of the expression of one of the spots (655 or ALDH1A3), in the six gels analyzed, between the cells transfected with miR-187 or with the negative control. The average fold change between the two conditions was -1.06 with a p-value of 0.003. ALDH1A3, aldehyde dehydrogenase family 1 member A3

In order to identify potential targets of the miR-187 a series of proteomic analysis and validation experiments was performed. miR-187 miRNA mimic was reintroduced in PC-3, LNCaP and DU-145 and demonstrated that miR-187 expression was recovered in PC-3, LNCaP and DU-145 cells (Fig 1B). A global proteomic approach using DIGE and LC-MS/MS was conducted with samples from PC-3 cells transfected with a negative control and PC-3 transfected with miR-187 miRNA mimic. Cells were harvested 72h after transfection, lysed and separated by bidimensional electrophoresis. After separating the protein extracts and fluorescence scanning, 9 differentially expressed spots were detected (Fig 2A). These 9 protein spots (p<0.05) displayed at least 1.06 fold regulation over six independent experiments. Seven out of these 9 spots showed a down-regulated expression upon miR-187 re-introduction, which was consistent with the expected inhibitory effect of the miRNA on most of its targets (S1 Table). Among these targets aldehyde dehydrogenase 1A3 (ALDH1A3) was identified (Fig 2B).

### 3.3. Validation of *ALDH1A3* as putative miR-187 target

**Fig 3:**
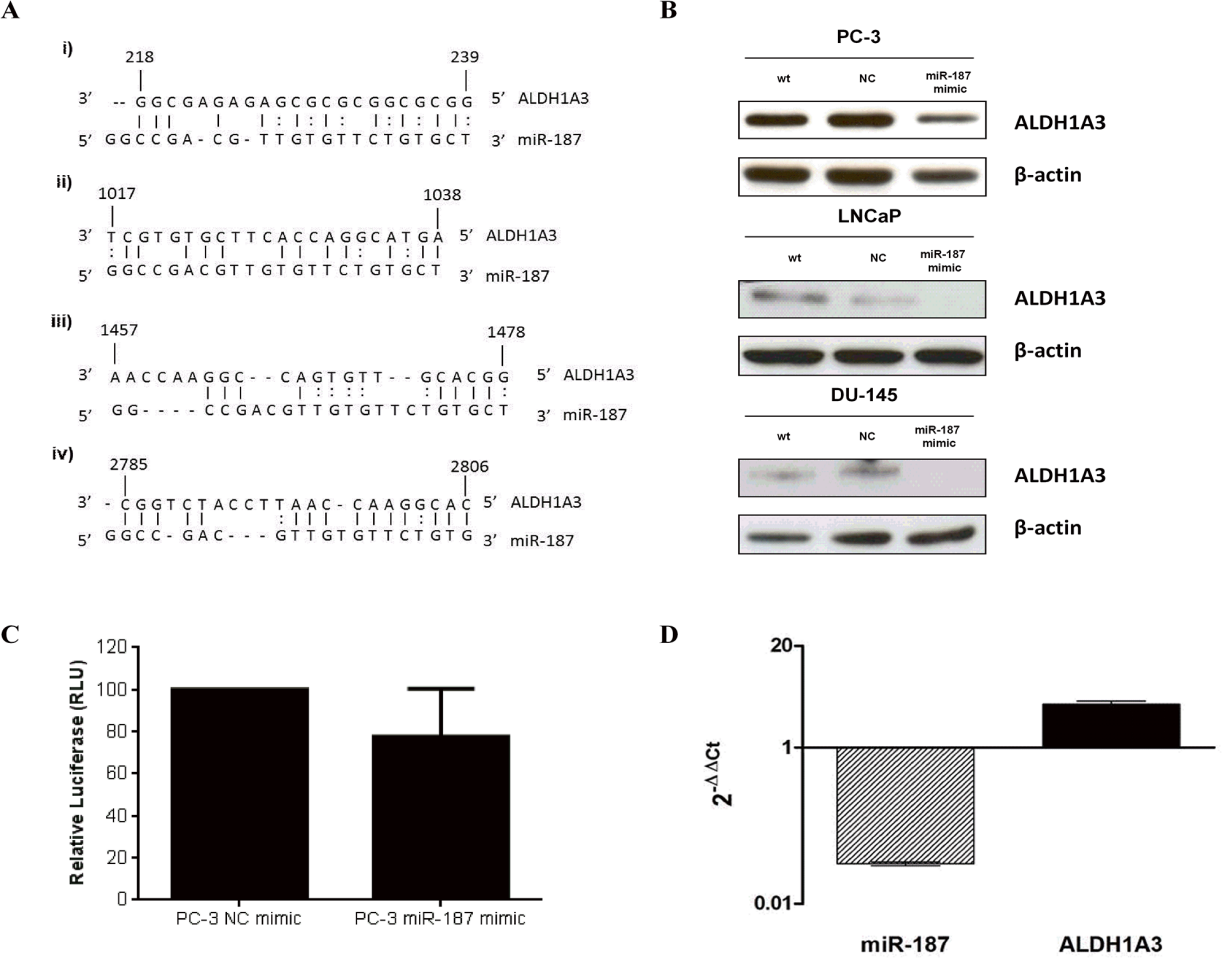
Validation of ALDH1A3 as target of miR-187. A) RNA22 predicted mRNA-miRNA heteroduplexes. RNA22 v1.0 software predicted three different miR-187 binding sites within ALDH1A3 mRNA sequence. Putative miR-187 binding sites were found in ALDH1A3 5’UTR region (i), coding region (CDS) (ii and iii) and 3’UTR (iv). All of them accomplish the criteria of a base pair minimum value of 14 and a maximum folding energy of -25. B) Western Blot analysis shows a reduction in the expression of the protein ALDH1A3 upon the re-introduction of miR-187 (miR-187 miRNA mimic transfected cells), confirming the inhibitory effect of the miRNA through its putative target. Cells were transfected with miR-187 miRNA mimic or negative control and harvested after 72h. Total cell extracts were prepared and electrophoresed by SDS-PAGE, followed by immunoblotting with anti-ALDH1A3 antibody. To ensure equal protein loading the membrane was immunoblotted with anti β-actin antibody. C) Luciferase reporter assay performed with firefly luciferase under control of the ALDH1A3 3′ UTR confirm the inhibition of ALDH1A3 mRNA expression (20% decrease of luciferase signal) upon miR-187 re-introduction. Data are presented relative to the vector control assigned a value of 100. The mean ± s.e.m. of three independent experiments is shown. D) Overexpression of ALDH1A3 mRNA was confirmed in a cohort of human prostate tumors (n = 96). There was an inverse correlation (not statistically significant) between the down-regulation of miR-187 found in these samples and the up-regulation of ALDH1A3.

To demonstrate that *ALDH1A3* is a target of miR-187, bioinformatics target screening; using the most common miRNA target prediction software (Targetscan, Pictar and miRanda) was performed. However, none of these programs matched the 3’-UTR region of *ALDH1A3* with the miR-187 sequence. Nevertheless, applying the RNA22 tool, which does not rely upon cross-species conservation, is resilient to noise and allows G:U pairing of target mRNA to miRNA seed sequence [13], confirmed a match between *ALDH1A3* and the miR-187 seed sequence (Fig 3A).

To validate these findings, we confirmed the strong down-regulation of ALDH1A3 upon miR-187 re-introduction by western blot analysis in PC-3, LnCaP and DU-145 cells (Fig 3B).

To further confirm the role of *ALDH1A3* as a miR-187 target a luciferase reporter plasmid containing the 3’UTR sequence of *ALDH1A3* was cloned into PC-3 cells transfected with miR-187 mimic. Enhanced expression of miR-187 (PC-3 miR-187 mimic) significantly reduced reporter activity of 3’UTR *ALDH1A3* constructs to about 20% compared with the control (PC-3 NC mimic) (Fig 3C).

qRT-PCR data showed a higher expression of *ALDH1A3* (1.2 fold) when compared to the samples re-expressing miR-187 (data not shown). Furthermore, we compared the expression of *ALDH1A3* mRNA expression with the down-regulation of miR-187 in two independent cohorts of primary PCa tumors from FFPE (n = 96) (Fig 3D) and fresh tissue (n = 10) (S1 Fig). However, no correlation between *ALDH1A3* and clinicopathological parameters was found, and then as expected, *ALDH1A3* did not constitute a prognostic indicator for either BPFS (p = 0.773) or PFS (p = 0.430) in this series.

**Fig 4:**
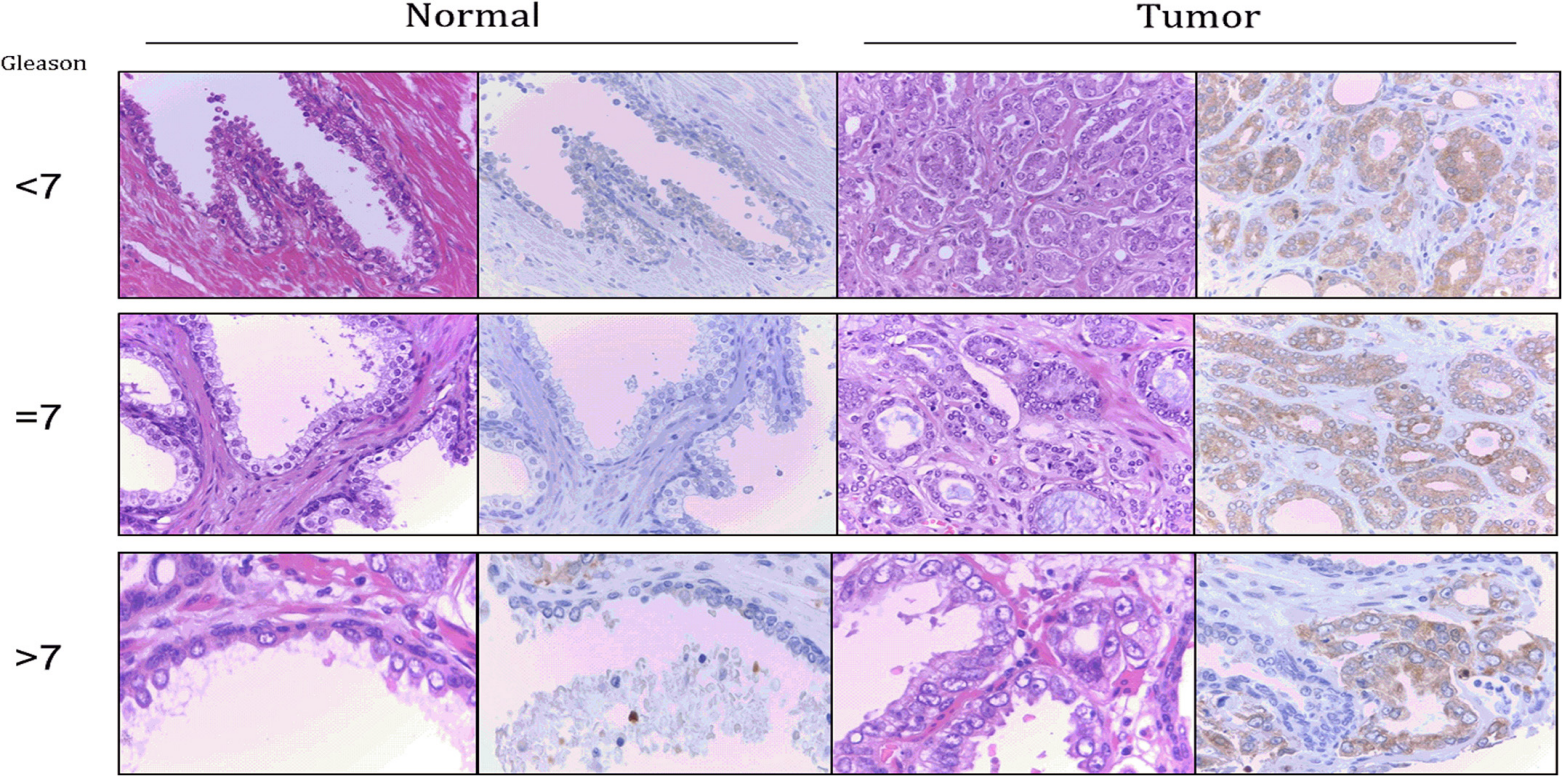
ALDH1A3 immunohistochemistry. Immunohistochemical staining for ALDH1A3 is significantly higher (p<0.0001) in tumor samples (right) than in normal controls (left). Staining between different tumor grades (Gleason score lower than 7, equal to or higher than 7) was compared finding a direct correlation between Gleason score and ALDH1A3 expression (p = 0.05). Immunohistochemical staining is shown for ALDH1A3 (right column) in each sample together with the hematoxylin & eosin-stained tissue (left column).

ALDH1A3 protein expression was further evaluated by immunohistochemistry (IHC) in the 195 cases included in the TMA (Fig 4). We found that ALDH1A3 was significantly (p<0.0001) up-regulated in PCa samples (average intensity = 1.42) when compared with normal prostate tissue (average intensity = 0.12). Moreover, in order to investigate the role of *ALDH1A3* as a miR-187 target, we studied the correlation with miR-187 expression. Interestingly, we found that miR-187 expression was significantly inversely correlated (p<0.0001) with ALDH1A3 protein expression. We further studied the correlation of *ALDH1A3* with clinicopathological parameters and prognosis. Although ALDH1A3 expression did not constitute a prognostic indicator, we found a statistically significant direct correlation with Gleason score (p = 0.05). Hence, 37% of cases with a Gleason score <7 showed high ALDH1A3 intensity of staining compared with 56% of PCa with Gleason≥7.

**Fig 5:**
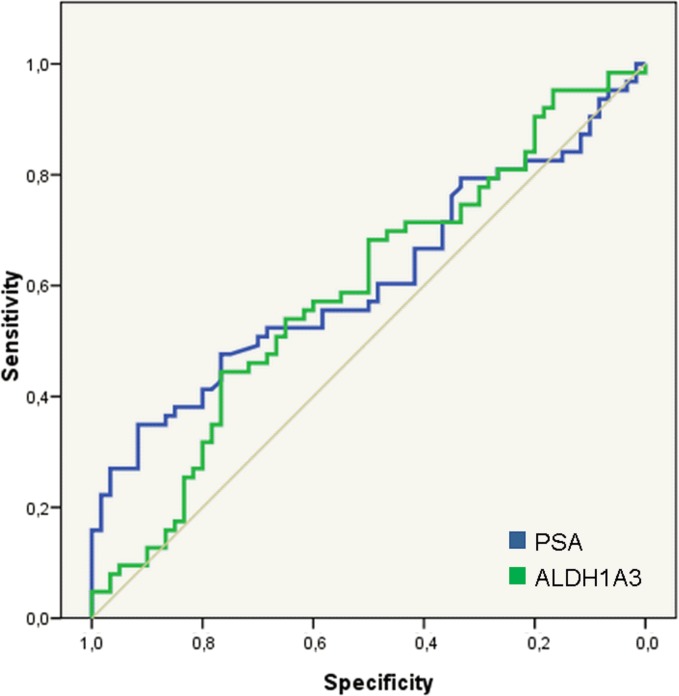
Diagnostic role of ALDH1A3 in urine samples. ROC curves from PSA and ALDH1A3 for predicting PCa in urine samples. The areas under the curve are 0.610 (95%CI 0.509–0.710; p = 0.036 and 0.591 (95% CI 0.490–0.692; p = 0.083) respectively. At a significance level of 10%, both PSA and ALDH1A3 were significantly associated with a positive biopsy of PCa.

To further explore the role of *ALDH1A3* in the diagnostic setting we performed an ELISA assay to measure ALDH1A3 expression in urine (n = 123). A univariate logistic regression model was performed to evaluate the predictive capability of this biomarker, together with PSA, for the presence of PCa in diagnostic biopsies. At a significance level of 10%, PSA and ALDH1A3 were both significantly associated with a positive biopsy of PCa (Fig 5).

## Discussion

miRNAs are predicted to control the activity of approximately 60% of all protein-coding genes, and have been shown to participate in the regulation of several cellular processes. By base pairing to mRNAs, microRNAs mediate translational repression or mRNA degradation [4]. Having previously demonstrated the role of miR-187 in PCa progression and diagnosis [8], we decided to further investigate potential targets of this miRNA that could be also of interest as biomarkers. For this purpose we reintroduced miR-187 precursor in PC-3 cells and performed a proteomic approach.

Sequences recognized by miRNA seeds are found in many genes, which makes it difficult to identify physiologically relevant miRNA–target relationships from sequence analysis alone [14]. Moreover, previous results obtained by Yang et al. [6] and Schramedei et al. [15] confirmed that less than 10% of proteins identified by a proteomic approach were predicted by commonly used algorithms such as Pictar, Targetscan and miRanda. In line with these findings, none of the proteins identified by proteomic screening in the present study were predicted *in silico* by the above mentioned algorithms. Nevertheless, using the RNA22 tool it was possible to find a match between miR-187 and our potential target in the *ALDH1A3* coding region (CDS). This algorithm is different from previously reported methods in that it does not use a cross-species sequence conservation filter, thus allowing the discovery of microRNA binding sites that may not be present in closely related species [13]. Moreover, RNA22 takes into account the hypothesis that, in addition to 3’UTRs, numerous binding sites are likely to exist in 5’UTRs and CDSs allowing the identification of previously unidentified miRNA/mRNA heteroduplexes.

In this study, 9 putative targets of miR-187 were identified by 2D-DIGE and MS analysis (S1 Table). From these we selected aldehyde dehydrogenase 1A3 (*ALDH1A3*) for further evaluation because it has been described to be regulated by androgens [16]. Western blot analysis, qRT-PCR and IHC confirmed the direct regulation of *ALDH1A3* by miR-187. First, the inverse correlation between ALDH1A3 and miR-187 was confirmed by recovering miR-187 expression in PC-3, LNCaP and DU-145 cells, which led to a down-regulation of ALDH1A3 protein levels. Second, the inhibitory effect of miR-187 on ALDH1A3 expression was further confirmed by a luciferase reporter assay that showed a decrease in ALDH1A3 expression (∼20% reduction in luciferase signal) upon miR-187 mimic transfection. Third, the inhibition of *ALDH1A3* was observed when analysing a cohort of PCa human patient samples, both fresh and FFPE tissues. In these cohorts, the strong down-regulation of miR-187 was accompanied by an increased *ALDH1A3* mRNA expression. Forth, the role of *ALDH1A3* as miR-187 target was confirmed by IHC analysis. Hence, ALDH1A3 was found to be up-regulated in prostate tumors and the expression of this protein is inversely correlated with the expression of the miRNA. In addition, the potential role of *ALDH1A3* as candidate prognostic biomarker for PCa was evaluated, although in the cohort of samples analysed it did not provide any additional information. Nevertheless, the association of *ALDH1A3* expression with Gleason score provides evidence of an increase in *ALDH1A3* expression with tumor staging. We have previously postulated that loss of miR-187 during PCa progression could indicate a role as tumor suppressor [8]. Additionally, ALDH1A3 was found to cooperate with PSA in the prediction of the biopsy result. Apart from its association with the presence of the tumor in IHC of FFPE slides, we were able to measure ALDH1A3 in urine samples, finding a positive association with tumor appearance. In this context, the identification of *ALDH1A3* as a miR-187 target and its up-regulation in PCa indicates its potential role as an oncogene with an implication in PCa development.

*ALDH1A3* is a member of the human aldehyde dehydrogenase family that includes different subtypes *ALDH1A1*, *ALDH1A2*, *ALDH1A6*, etc. that catalyze the oxidation of retinal to retinoic acid (RA)[17], which is required for normal prostate development [18]. The implication of these enzymes in RA synthesis causes them to function as key enzymes in pathways associated with cell proliferation, differentiation and survival. *ALDH1A3* has been found to play a role as a predictor of metastasis in breast cancer [19]. ALDH1A isozymes, mainly ALDH1A1 and ALDH1A3, have been also described as markers of cancer stem cells in different tumors and key determinants for the survival and drug resistance of cancer cells [19, 20]. In agreement with the association with stemness of ALDH1A3, miR-187 has been recently identified as an miRNA that specifically characterizes human embryonic stem cells and induces pluripotent stem cells [21]. Therefore both genes, miRNA and target, seem to regulate pluripotent cell characteristics which are related with a more undifferentiated and aggressive tumor phenotype. In this regard, recent results show that high ALDH activity can be also used to isolate human prostate cancer cells with significantly enhanced tumorigenicity and metastatic behavior [22]. Thus, using a FACS sorting kit such as ALDEFLUOR, which classifies cells according to ALDH activity, might be a useful tool for the stratification of prostate cancer patients at risk of developing metastatic disease.

It is recognized that a single miRNA can modulate several genes [4, 5] and probably the effects of the restoration of miR-187 are broader than those observed in a single gene. Nevertheless, our data illustrate for the first time the role of ALDH1A3 as a miR-187 target in PCa and provide insights into the utility of including this protein as a new biomarker for PCa.

## Supporting Information

S1 Fig ALDH1A3 expression in an independent cohort of PCa fresh tissue. With the aim of performing a validation in an independent set of PCa patients, overexpression of ALDH1A3 mRNA was confirmed in a cohort of human fresh prostate tumors (n = 10). There was an inverse correlation (p<0.0001) between the down-regulation of miR-187 found in these samples and the up-regulation of ALDH1A3.
(TIF)

S1 Table Putative miR-187 predicted targets. (DOCX)
