# Supplementary figures and images for: MiR-187 Targets the Androgen-Regulated Gene ALDH1A3 in Prostate Cancer
Source: PLoS One. 2015 May 13;10(5):e0125576. doi: 10.1371/journal.pone.0125576 (PMC4430273; doi:10.1371/journal.pone.0125576)

S1 Table. Putative miR-187 target predicted by DIGE-LC/MS


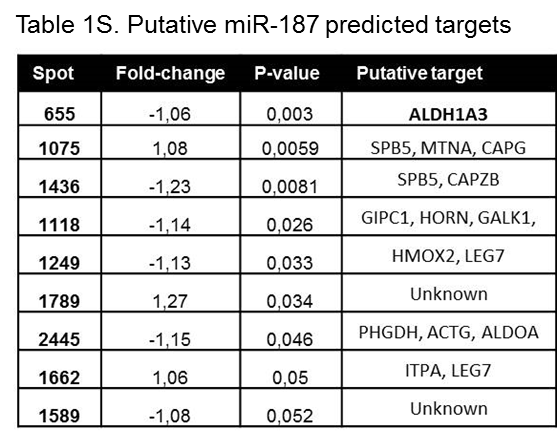

Supplement: S1 Table — (DOCX) [file pone.0125576.s002.docx]
